# Global kidney health priorities—perspectives from the ISN-GKHA

**DOI:** 10.1093/ndt/gfae116

**Published:** 2024-05-20

**Authors:** Ikechi G Okpechi, Valerie A Luyckx, Somkanya Tungsanga, Anukul Ghimire, Vivekanand Jha, David W Johnson, Aminu K Bello

**Affiliations:** Division of Nephrology and Immunology, Faculty of Medicine and Dentistry, University of Alberta, Edmonton, Alberta, Canada; Division of Nephrology and Hypertension, Department of Medicine, University of Cape Town, Cape Town, South Africa; Department of Public and Global Health, Epidemiology, Biostatistics and Prevention Institute, University of Zurich, Switzerland; Renal Division, Brigham and Women's Hospital, Harvard Medical School, Boston, MA, USA; Department of Paediatrics and Child Health, University of Cape Town, South Africa; Division of Nephrology and Immunology, Faculty of Medicine and Dentistry, University of Alberta, Edmonton, Alberta, Canada; Division of General Internal Medicine-Nephrology, Department of Medicine, Faculty of Medicine, Chulalongkorn University, Bangkok, Thailand; Division of Nephrology and Immunology, Faculty of Medicine and Dentistry, University of Alberta, Edmonton, Alberta, Canada; Division of Nephrology, Department of Medicine, University of Calgary, Calgary, Alberta, Canada; George Institute for Global Health, University of New South Wales (UNSW), New Delhi, India; School of Public Health, Imperial College, London, UK; Prasanna School of Public Health, Manipal Academy of Higher Education, Manipal, India; Centre for Kidney Disease Research, University of Queensland at Princess Alexandra Hospital, Brisbane, Australia; Translational Research Institute, Brisbane, Australia; Department of Kidney and Transplant Services, Princess Alexandra Hospital, Brisbane, Australia; Division of Nephrology and Immunology, Faculty of Medicine and Dentistry, University of Alberta, Edmonton, Alberta, Canada

**Keywords:** health finance, health policy, kidney replacement therapy, kidney transplantation, workforce

## Abstract

Kidney diseases have become a global epidemic with significant public health impact. Chronic kidney disease (CKD) is set to become the fifth largest cause of death by 2040, with major impacts on low-resource countries. This review is based on a recent report of the International Society of Nephrology Global Kidney Health Atlas (ISN-GKHA) which uncovered gaps in key vehicles of kidney care delivery assessed using World Health Organization building blocks for health systems (financing, services delivery, workforce, access to essential medicines, health information systems and leadership/governance). High-income countries had more centres for kidney replacement therapies (KRT), higher KRT access, higher allocation of public funds to KRT, larger workforces, more health information systems, and higher government recognition of CKD and KRT as health priorities than low-income nations. Evidence identified from the current ISN-GKHA initiative should serve as template for generating and advancing policies and partnerships to address the global burden of kidney disease. The results provide opportunities for kidney health policymakers, nephrology leaders and organizations to initiate consultations to identify strategies for improving care delivery and access in equitable, resource-sensitive manners. Policies to increase use of public funding for kidney care, lower the cost of KRT and increase workforces should be a high priority in low-resource nations, while strategies that expand access to kidney care and maintain current status of care should be prioritized in high-income countries. In all countries, the perspectives of people with CKD should be exhaustively explored to identify core kidney care priorities.

## INTRODUCTION

Kidney diseases are a serious and under-recognized global epidemic. At least 10% of the world's population have kidney diseases [1]. Chronic kidney disease (CKD) is the third fastest growing cause of death globally, the only non-communicable disease (NCD) with year-on-year increase in age-standardized mortality rates, and is set to become the fifth largest global cause of death by 2040 [2]. Despite these worrisome statistics and the fact that CKD accounted for 1.2 million deaths and 35.8 million disability-adjusted life years in 2017 [3], it is only recognized as a health priority in 48% of countries worldwide [4]. With the rising public health threat of CKD, global nephrology leaders developed four key strategies to tackle CKD: (i) early CKD and risk factors identification, (ii) understanding causes and outcomes of CKD, (iii) improving CKD outcomes and (iv) developing new therapeutic strategies (Table 1) [5]. These strategies may need to be tailored and implemented differentially across countries, as priority-setting for policy and care may differ between jurisdictions [6]. For instance, while early disease identification and prevention strategies should be highly prioritized in all countries, low-resource countries with limited availability and access to kidney care should place more value on preventive strategies which can lead to improved kidney outcomes.

**Table 1:** The ISN recommendations for Global Kidney Health.

Theme	Sub-themes	Suggested strategies to achieve kidney health priority
Improve the identification of CKD and reduce risk factors for CKD	Work toward kidney disease prevention, early diagnosis and treatment	● Ensure healthy adult, maternal and child lifestyles and nutrition
		● Reduce the burden of co-occurring conditions (e.g. diabetes, hypertension, cardiovascular diseases and/or obesity)
		● Reduce unhealthy addictions (e.g. drugs, tobacco, alcohol)
		● Promote strategies that include medications use, KRT, promote recovery and prevent disease recurrence
		● Prevent and respond to epidemic and endemic infectious diseases that impact kidney health
	Monitor kidney disease burden	● Implement and support surveillance programs to better understand the burden of AKI and CKD
		● Develop robust national and regional registries on the incidence, prevalence and aetiology of AKI, CKD and kidney failure with or without KRT
	Raise public awareness	● Improve health literacy on kidney disease and its risk factors to empower patients in decision-making and self-management of their conditions.
Improve the understanding of causes and consequences of CKD	Address kidney diseases across the life course through UHC	● Enabling UHC ensures access to quality health services, including health promotion, rehabilitation, treatment, and palliative care without financial burden throughout their life-course (this should begin with universal access to antenatal care and child health visits, sustainable access to effective and affordable quality diagnostics and medications for detecting and treating risk factors, such as hypertension, diabetes, cardiovascular and urologic diseases)
	Integrate kidney disease services within existing health and multisectoral initiatives	● Pursue the 2030 SDG
		● Prioritize UHC
		● Utilize the life course approach to chronic diseases
		● Use the ‘recovery to build back better’ for NCDs
	Improve education on kidney diseases among all healthcare workers	● Ensure that a lack of clinical awareness among health workers is not a barrier towards optimal access to tools and medications needed for protection, diagnosis and treatment of kidney disease
Improve outcomes with current knowledge	Strengthen the kidney care workforce	● Support education (on screening, prevention and treatment of kidney disease) and the growth of a skilled nephrology workforce to ensure the highest level of patient care across the spectrum of kidney diseases
	Strive for equitable and sustainable access to care for kidney failure	● Strategies to ensure early diagnosis of kidney disease
		● Strategies that ensure availability and access to appropriate treatments
		● Access to KRT
Develop and test new therapeutic strategies	Promote and expand kidney transplantation programs	● Promote and expand kidney transplant programs that uphold ethical practice in management of all donors and recipients, adhere to the principles of the Declaration of Istanbul and ensure equal access to transplantation regardless of an individual's income
	Support research for kidney diseases	● Promote and support publishing research work in mainstream journals.
		● Increase and support human resources in academic and medical workforces to advance the research and treatment of kidney diseases

SDG, sustainable development goals.

In high-income countries (HICs), which have well-developed programs for management of advanced kidney disease, the high costs associated with kidney care and the use of public funds for coverage has led several programs to focus on cost reduction, alongside enhancing population health management capabilities, improving quality measurement and advancing health equity [7]. On the other hand, because of competing demands on limited funding and non-availability of resources in low-income countries (LICs) and lower-middle-income countries (LMICs), early identification and management of CKD and its risk factors (such as hypertension, diabetes and nephrotoxic agents) rather than development of dialysis services have been suggested as higher priorities to curb the burden of kidney diseases, improve quality of life and reduce healthcare costs [8].

**Figure 1:**
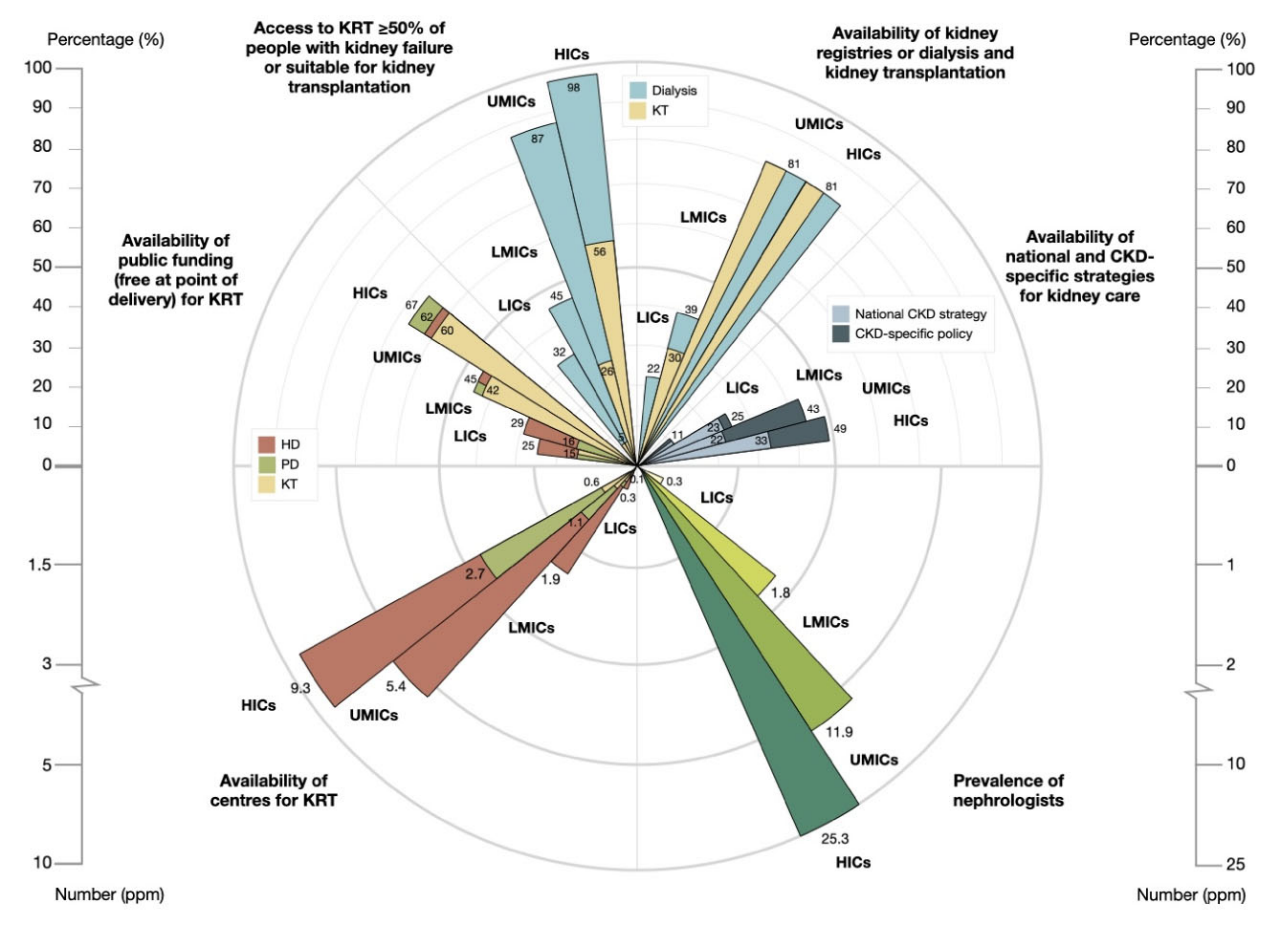
Summary of key findings in the 2023 ISN-GKHA survey by country income levels. Availability of public funding (free at point of delivery) for KRT; access to KRT (dialysis and kidney transplantation) for ≥50% of people with kidney failure or suitable for kidney transplantation; availability of kidney registries for dialysis and transplantation; national and CKD-specific strategies for kidney care; prevalence of nephrologists; and availability of centres for KRT. KT, kidney transplantation; PD, peritoneal dialysis.

In a recent joint statement from the three major international nephrology organizations [9], a case was made for the recognition of kidney diseases by the World Health Organization (WHO) as a major driver of premature mortality. To achieve this, unmet policy, advocacy and implementation requirements were identified as priorities in order to alleviate the burden of kidney disease including (i) improved access to care, (ii) better prevention strategies, (iii) developing and scaling up novel balanced models of care, (iv) greater awareness and education, (v) addressing social determinants of kidney health, (vi) increased funding for research and development, (vii) international cooperation and coordination, and (viii) greater engagement with patient communities [9]. The recently published International Society of Nephrology Global Kidney Health Atlas (ISN-GKHA) provides a comprehensive state of care for provision of kidney services in all world regions (167 countries covering 97.4% of the world population across all World Bank income groups) (Fig. 1) [4]. The high-level findings demonstrate that the people living in HICs had more services for kidney replacement therapies (KRT), greater access to KRT, more public funds allocated to KRT, larger workforces, more robust health information systems, and greater government recognition of CKD and KRT as health priorities compared with LICs and LMICs. In this review, we leverage information from the recent iteration of the ISN-GKHA to discuss global kidney health priorities. Our review is therefore structured around the WHO building blocks for health systems, emphasizing key areas of focus for optimal delivery of kidney health across country income groups.

## FINANCING KIDNEY CARE

The cost of all CKD care is enormous, exceeding those of cancer or diabetes care, and was estimated to exceed €140 billion annually in Europe [10]. Financing for kidney care remains highly variable and often challenging both within and between countries. The 2023 iteration of the ISN-GKHA reported that kidney care was publicly funded and free at the point of delivery in only 27% of countries for non-dialysis CKD treatment, 44% for acute kidney injury (AKI), 45% for maintenance haemodialysis (HD), 42% for continuous ambulatory peritoneal dialysis (CAPD), 53%–58% for dialysis access creation, 51% for kidney transplant surgery and 36% for kidney transplant medications (Fig. 1) [4]. Given that KRT is expensive and responsible for the highest amount of catastrophic healthcare expenditure, it is concerning that Universal Health Coverage (UHC) for all aspects of KRT was only available in 49% of countries, ranging from 13% in LICs to 18% in LMICs to 51% in upper-middle-income countries (UMICs) to 75% in HICs [4]. When comparing LICs with HICs, government and out-of-pocket health spending per person varied 182- and 42-fold, respectively. Out-of-pocket spending on HD exceeded 75% of the total care cost in 20 (12.2%) countries and accounted for 100% of HD costs in seven countries (Democratic Republic of the Congo, Ghana, Madagascar, Nigeria, Tajikistan, Papua New Guinea and Vanuatu). When UHC is not provided for kidney care, catastrophic out-of-pocket health expenditure, defined as an amount exceeding 10% of household income, becomes more common and is associated with excessive mortality, particularly in vulnerable and disadvantaged populations [11, 12]. For example, in a systematic review of 68 studies of outcomes in 24 456 adults and 809 children with kidney failure requiring dialysis in sub-Saharan Africa, 96% of adults and 95% of children were known or presumed to have died because they could not afford care [13]. An earlier global systematic review had shown that at least 2.5 million people might have died in 2010 due to lack of access to KRT with most deaths linked to lack of affordability of treatment [14].

Whilst there is an exigent need to provide affordable kidney care in low-resource settings due to a growing burden of CKD [15, 16], governments need to make evidence-informed decisions on what aspects of kidney care to prioritize, balanced against other competing priorities such as other NCDs, infectious diseases, maternal and child mortality, and environmental health [12]. The ISN has put forward a framework for establishing integrated kidney care programs in LICs and LMICs, which accords highest priority to programs targeting improved awareness, risk factor mitigation, early detection and prevention of progression of CKD, and which may be most efficient when integrated with other NCD prevention programs [6, 17]. Such programs are likely to be highly effective and cost-efficient compared with those focused on treating kidney failure given the findings from an Australian study which showed that funding CKD detection and prevention programs provided an AUD $45 return on every AUD $1 invested [18]. However, it is unclear whether this benefit can be realized in low-resource settings due to differences in health system capacity. The next highest financing priority for resource-limited countries is conservative kidney management (CKM) programs to delay disease progression, minimize complications, relieve symptoms, and provide emotional, psychological and spiritual support to patients and their families [17, 19]. Whilst KRT is expensive, kidney transplantation offers the best outcomes at the lowest costs, although it still needs to be supported by at least a limited dialysis program. LICs and LMICs choosing to fund KRT programs need to consider innovative approaches to achieving better outcomes at reduced costs, such as shared transplant infrastructure (like the successful partnership program between Iran, Tajikistan and Azerbaijan) [6], PD first programs (as exemplified in Thailand and Hong Kong) [20], affordable dialysis projects [21], implementation of guidelines to maintain clinical care standards, efficient use of the kidney workforce (e.g. telehealth, task substitution, etc.) [17, 22], establishment of robust procurement mechanisms to lower healthcare costs, utilization of evidence-based priority-setting tools [23] and embracing public–private partnerships [24].

The ISN-GKHA showed that more HICs (especially in Europe) than countries in other income categories use public funds to reimburse kidney care. The challenge in such settings is to identify not only ways of lowering current cost of care [25], but also how to increase funding to combat the epidemic of CKD and fund ambitious projects such as the target to reduce CKD incidence by approximately 10% by 2029 [26, 27]. Such targets can be achieved through increased financial investments for programs that increase awareness, prevent risk factors, improve early identification and interventions, and increase research and innovation in kidney care. Such innovations may include solutions for minimizing greenhouse gas emissions (which are 70-fold higher in HICs than LICs) [28], water consumption, waste production and management, as well as increasing energy-efficient care, low-cost manufacturing of elements of dialysis, and the promotion and increased utilization of telemedicine for care delivery [29]. For example, increased funding to develop or expand telemedicine has potential to improve access to healthcare, especially in remote or rural areas of HICs or in LICs and LMICs where the kidney care workforce is inadequate, for improving CKD awareness and for increasing adoption of best practice guidelines for CKD care [30]. Increased funding for research will lead to improved quality of clinical trials and increase the chances of developing novel diagnostics and therapeutics and more quickly achieving translation from ‘bench to bedside’ [26]. Innovations with low environmental impact should be prioritized.

## INCREASING AVAILABILITY OF KIDNEY CARE SERVICES

Kidney care services for providing KRT and CKM are integral to the continuum of care for patients living with advanced stages of CKD [31]. KRT is technology-driven and consumes a significant proportion of healthcare costs across nation states, often consuming 2%–3% of healthcare budgets in the HICs for around 0.1% of the population [32]. In the 2023 ISN-GKHA report, chronic HD was reported to be available in almost all participating countries (100% of UMICs and HICs, 96% of LMICs and 95% of LICs) [4] (Fig. 1). Similarly, CAPD services were available in 130 (79%) participating countries, ranging from 21% of LICs to 97% of HICs, while kidney transplantation services were reported in 116 (70%) countries, ranging from 21% of LICs to 86% of HICs. Of interest, CKM through shared decision making was also available in a higher proportion of HICs (72%) compared with LICs (42%) and LMICs (33%). Although kidney transplantation should have the highest priority when deciding which KRT modality to make available, kidney transplantation services depend on effective pre-dialysis and dialysis services in order to be successful [6]. However, LICs and LMICs should prioritize CAPD which has several advantages over HD, including greater technical simplicity, lesser need for trained staff, greater cost-effectiveness and possibly better survival [33]. Moreover, efforts to increase locally manufactured CAPD dialysate within low-resource nations can significantly reduce costs and expand access to care [34]. In HICs, there is also a need to expand the practice and utilization of home hemodialysis (HHD) services given the number of clinical, survival and economic advantages associated with home therapies [35]. Despite these advantages, the global prevalence of HHD use remains low [35]. Data from Europe indicate that HHD is available in a few countries including Belgium, Denmark, Finland, France, Norway, Serbia, Spain, Sweden, Switzerland, the Netherlands and the UK, with the highest adjusted prevalence reported in Denmark (28.3 pmp) [36]. Efforts to expand HHD access should be pursued while addressing logistical, educational or financial barriers [37].

Another important component of KRT care provision is accessibility of the services, given that availability does not mean accessibility due to a host of factors, including economic (funding), geographic (rural/remote location) or political/policies (e.g. KRT rationing in South Africa) [38]. The ISN-GKHA reported that, in 74% of countries with available dialysis (HD and CAPD) services, more than half of people needing dialysis could access treatment at the onset of kidney failure [4]. Access to KRT, defined as ability to receive treatment when needed, increased with rising country income levels, ranging from 32% in LICs to 98% in HICs. One study reported that of the 40 functioning HD centres in Ghana, 33 were located in 2 of the 16 regions serving 35.2% of the entire population (predominantly urban), 7 regions had one centre each, while the remaining 7 regions had no treatment centres [39]. In addition, of the 40 centers, 27 were private. Such observations indicate significant disparities in access to services that preferentially favour urban dwellers and those with the ability to pay out of pocket. Governments in low-resource countries should ensure equitable distribution of these services such that kidney care can be readily accessed in rural as well as urban centres [13, 39]. Transparent priority setting involving all stakeholders, including people with kidney diseases, is required to equitably enhance KRT coverage, although this will take time [23]. Enabling policies and government buy-ins are significant factors affecting KRT services uptake across countries and regions. Major stakeholders, including national governments, international organizations and nongovernmental organizations (NGOs), have a corporate responsibility to support KRT access, particularly in LICs, to improve equity of access and quality care [40].

## INCREASING AVAILABILITY OF ESSENTIAL MEDICATIONS AND EQUITABLE ACCESS TO KRT

The majority of people living with CKD are in stages 1–3 [3]. This fact makes early diagnosis an imperative as timely interventions with a combination of medications and optimization of lifestyle can slow disease progression, and prevent or delay dialysis requirement, cardiovascular complications and death [41, 42]. Despite mounting evidence of unprecedented benefits of newer therapeutic strategies, such as sodium-glucose cotransporter-2 (SGLT2) inhibitors, their uptake remains suboptimal globally [43, 44]. Many factors contribute to this observation, including lack of prioritization of CKD at the policy level, lack of universal access to affordable and quality primary care, lack of awareness or training among all cadres of healthcare workers, lack of access to appropriate diagnostics and medications, as well as insufficient patient engagement and education to ensure appropriate initiation, adherence and persistence of CKD therapies [45]. A holistic approach is required to tackle all barriers systematically to ensure that the right people are screened at the right time and are treated with the right medications over their lifetimes.

**Figure 2:**
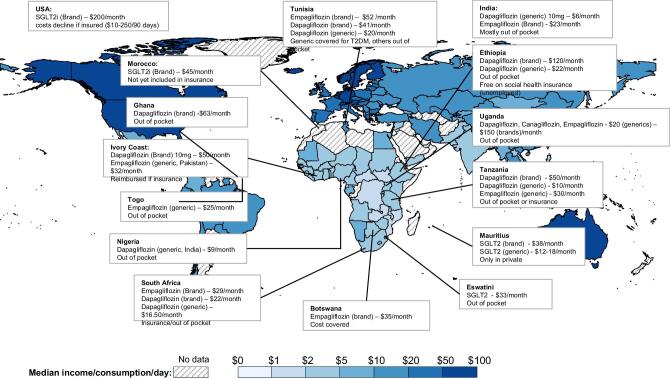
Global distribution of median income or consumption per day (2019) and monthly costs of SGLT2 inhibitors across Africa. T2DM, type 2 diabetes mellitus; $, US dollar. (Reproduced with permission from ref. [48]).

Low-resource countries frequently have inconsistent availability and supply of essential medicines [46] due to various logistical barriers, including inefficient supply chains, lack of storage capacity, poor access to standard quality drugs and lack of generic medications [46]. A WHO study across 53 countries found that essential cardiovascular and hypertension medications, which are largely those required for treatment and prevention of CKD, were less available in low-income settings, more available in private settings, with the cost for a single brand medication equivalent to 6 days’ wages while that for a generic brand was 1.8 days’ wages [47]. These prices are unaffordable for many people, with increased unaffordability when more medications are required, as is often the case for CKD. A recent survey of nephrologists in Africa regarding the costs of SGLT2 inhibitors also revealed significant price disparities across countries and between brand and generic medications, with out-of-pocket costs being several folds higher than the median income in many countries (Fig. 2) [48].

The WHO has put forward an access to drugs and medicine framework, which incorporates four essential pillars: rational medication use, affordable pricing, sustainable financing, and reliable health and supply systems [47]. These highlight the importance of health systems–wide approaches, including appropriate healthcare worker education, rational guideline development or modification for local context, appropriate policy development and resource allocation to include CKD, negotiation with pharma to embrace fair prices and fair profits [49], strengthening the quality of generics, combatting corruption, and progressive realization of UHC [50].

## EXPANDING KIDNEY CARE WORKFORCE TO MEET FUTURE DEMANDS

The ISN-GKHA reported a >80-fold difference in nephrologist densities between LICs (0.3 pmp) and HICs (25.3 pmp) and shortages of various cadres of the workforce, including transplant surgeons, dialysis nurses, dialysis technicians, etc. [4] (Fig. 1) The specific causes of workforce shortages differ between HICs and LMICs/LICs. Shortages in HICs are often driven by a lack of trainee interest arising from uncertainty in prospective employment or increasing interest in more lifestyle-friendly specialties [51]. In contrast, workforce retention in LICs and LMICs is frequently hindered by poor healthcare infrastructure, poor remuneration, geopolitical tensions and unsafe working conditions [52].

Region-specific barriers need to be addressed to enable workforce expansion. As training opportunities are often lacking in low-resource settings, collaboration based on the ISN Fellowship program can be built with other major nephrology organizations such as the European Renal Association and the American Society of Nephrology for training opportunities for physicians from LICs where such need is greatest [53]. Task-shifting strategies, improved quality of training for nurses, pharmacists and allied kidney care workforce, as well as strategies that address excessive workforce migration to HICs need to be addressed to expand the workforce in low-income settings [54]. In HICs where interest in nephrology among medical students and internal medicine residents is low [51], innovative care models including environmentally sustainable kidney care [e.g. Global Environmental Evolution in Nephrology and Kidney Care (GREEN-K initiative)] [55], novel models of care (e.g. wearable and implantable systems) [56], and use of novel technologies and artificial intelligence in care have the potential to attract trainees and improve care delivery [57].

## STRENGTHENING RELIABLE KIDNEY DISEASE INFORMATION SYSTEMS

Kidney registries, integral components of Health Information Systems, play crucial roles in identifying regional/national disease epidemiology/outcomes/trends across diverse populations and social groups, auditing kidney care quality, generating hypotheses for clinical trials, and facilitating health service planning and healthcare policies [58]. According to the 2023 ISN-GKHA [4], registries for non-dialysis-dependent CKD, dialysis, kidney transplantation and CKM are available in 31 (19%), 102 (63%), 94 (58%) and 9 (6%) countries, respectively. However, most LICs lack kidney registries (Fig. 1).

A number of issues need addressing. At the national level, establishing a registry requires collaboration across multiple levels, involving the national nephrology society, advocacy groups and jurisdiction. This collaboration should extend to data sharing based on data ownership agreements. Secondly, the labor-intensive nature of data collection needs simplification. Utilizing online or mobile platforms with user-friendly interfaces can streamline the process and reduce workload. Simplifying the dataset itself makes the data collection more comprehensive. Thirdly, ensuring the validity and completeness of collected data is essential. Simplifying questionnaires by incorporating closed questions or list boxes instead of open-ended ones can enhance data quality [59]. Regular audits further contribute to maintaining data accuracy. Fourthly, the substantial workload involved in establishing and maintaining a registry highlights the need for incentives to support personnel. Adequate funding is essential to facilitate these efforts. Furthermore, to improve quality, international collaboration and support are crucial for overcoming these challenges globally. Low-resource countries with limited availability of kidney registries and capacity to characterize kidney disease burden can leverage the ISN's Sharing Expertise to support the set-up of Renal Registries (SharE-RR) toolkit [60] and the the QUality European Studies (Nephro-QUEST) [61] for setting up kidney registries that utilize high-quality standards and ethical processes. Such strategies can allow development of registries that enables comparison of information across countries and improves global understanding of disease epidemiology and outcomes. In addition, such processes will enable effective communication with all stakeholders including the public and policymakers [62].

## IMPROVING ADVOCACY AND GROWING FUTURE LEADERS IN NEPHROLOGY

Given the already high and rapidly growing burden of AKI and CKD worldwide, its disproportionate impact on disadvantaged people and societies, and the uniform neglect by the global health community, contextually specific advocacy for kidney diseases assumes a priority [3]. According to the 2023 ISN-GKHA, only 19%, 48% and 63% of governments worldwide recognize AKI, CKD and KRT, respectively, as health priorities [4]. As these data show, governments often prioritize KRT, to the neglect of early detection and disease prevention (Fig. 1).

Effective advocacy thrives on targeted communication. The target audience includes policymakers (arbiters of resource allocation and legislative landscapes who navigate a multitude of competing interests), healthcare professionals (who often do not appreciate the burden and consequences of kidney disease), public and civil society organizations (advocacy groups and NGOs often engaged in on-ground service delivery). Informed individuals make informed choices, and advocacy is critical to raising awareness and empowering communities to actively participate in shaping their health (Table 2). Data from LMICs show that almost 90% of people with CKD in communities are not aware of their disease [63]. Advocacy should amplify the voices of vulnerable populations that have a particularly high burden of kidney diseases. Advocacy groups focusing on AKI, CKD and kidney failure/KRT exist in 11%, 40% and 34% of countries, respectively [4].

**Table 2:** Championing kidney health—advocacy for a public health renaissance.

Strategies	Action
• Understand the audience	• Lobby policymakers
• Craft a clear message	• Educate healthcare professionals
• Highlight the human cost	• Identify allies, promote accessible, integrated care
• Emphasize the economic impact	• Empower communities
• Promote early detection and prevention with an equity lens	• Leverage communication channels
• Develop collaboration	• Amplify the voice of vulnerable populations
• Champion research and innovation	• Develop learning health systems
• Groom next generation of leaders and advocates	• Customize to local circumstances

Crafting a compelling narrative is critical. The message crafted for each stakeholder group must be succinct, data-driven and evidence-based, and resonate with their specific concerns. In particular, showcasing the economic and social ramifications of kidney diseases and cost–benefit analyses highlighting the long-term financial returns of prioritizing kidney health interventions can prove persuasive with policymakers. Additional strategies include humanizing the message through compelling narratives, weaving early detection and prevention strategies in existing disease management programs (e.g. diabetes, hypertension, cardiovascular disease) rather than standalone initiatives [64], and presenting actionable proposals that can be implemented using existing resources. Advocacy should call for easy access to essential diagnostics and therapeutics for identifying and managing kidney diseases at all healthcare system levels [46]. Where possible, health technology assessment supported by health economic studies should inform the development of approaches suited to local circumstances.

Advocacy is crucial in securing funding for critical research initiatives, promoting collaboration between public and private sectors, and advocating for ethical frameworks to guide research (Table 2). Encouraging investment in research on kidney disease prevention, diagnosis, and treatment paves the way for future advancements. Implementation research [65], co-produced with stakeholders and targeting outcomes of public health interest (acceptability, scalability, sustainability and affordability), should be prioritized.

Tangible actions are critical to galvanizing change. Strategies include policy dialogues, lobbying efforts, public hearings, written communication with policymakers, collaborating with media (traditional and social media and interactive web) platforms, community-focused awareness campaigns, and education targeted to healthcare professionals and support groups. By building coalitions across diverse stakeholder groups, including affected individuals, healthcare professionals, NGOs, civil society organizations and the public, kidney health advocates create a united front, amplifying messages and leveraging each other's strengths.

Young healthcare professionals hold immense potential to become influential voices for positive change. They bring fresh perspective and innovation, are technologically savvy, understand the value of entrepreneurship, can leverage digital platforms for effective communication, and are driven by idealism and a desire to make a difference, bringing passion and energy to advocacy. As they progress in their careers, young leaders will occupy prominent positions within healthcare systems and public health institutions, ensuring sustained focus on advocacy efforts.

Grooming effective advocates and future leaders requires training on public health advocacy, policy analysis, communication skills and social justice; providing mentorship opportunities by connecting them with experienced public health advocates and policymakers; organizing skill-building workshops; encouraging research focused on public health issues, allowing them to contribute to evidence generation and develop innovative solutions that inform advocacy efforts; and undertaking community engagement initiatives. Investing in developing young healthcare professionals as public health advocates creates a ripple effect. Their passion and skills not only contribute to specific advocacy campaigns but also inspire their peers and future generations to join the cause. These programs (such as the ISN's Emerging Leaders Program and the European Renal Association's Young Nephrologist Program) help to create a global network of early-mid career kidney health professionals to promote global kidney health.

## INCREASING PATIENTS’ VOICES AND ENGAGEMENT IN PRIORITY-SETTING

A core principle of the Alma-Ata Declaration of 1978 is the right and duty to participate in the planning and implementation of an individual's healthcare [66]. Patients are infrequently involved in healthcare decision-making, especially in LICs and LMICs, due to scarcity of services, low awareness, and the perception that their financial and emotional problems outweigh their illness [67]. In one survey of people receiving dialysis, 51.9% stated that the decision to initiate dialysis reflected their physicians’ preferences [68]. Patient engagement in healthcare delivery should involve inclusion of their voices in the design, implementation and delivery of care services and can be achieved using patient groups or advisory boards [69]. Increased patient engagement has the potential to improve person-centred care, shared decision-making, and reduce hospitalizations and cost of care [70]. Patients who participated in the recent ISN-GKHA identified essential medicines and KRT cost reduction as important strategies for improving kidney care. However, the ability to work, mobility and finances were the factors they identified that negatively impacted their well-being [4]. It is critical to include patients’ perspectives when developing policies for kidney care and clinical and research programs.

## CONCLUSION

Following the 2023 ISN-GKHA, the status of kidney care across countries and regions has become clearer as this global database continues to demonstrate significant gaps in care, especially in low-resource nations. Evidence identified from this initiative should serve as a template for generating and advancing priorities including policies and strategic partnerships to address the global burden of kidney disease. The results provide opportunities for all world regions to embark on consultations and dialogues to identify key gaps in care delivery and access to care in equitable, resource-sensitive manners. Low-resource countries should identify means of increasing the use of public funding for kidney care, lowering the cost of KRT, and retaining and increasing the kidney care workforce to improve outcomes. HICs should prioritize expanding access to KRT and developing new therapies for kidney care. In each country, the views of people living with kidney failure will be paramount in identifying key areas of policy prioritization and implementation.

## Data Availability

No new data were generated or analysed in support of this research.
